# No Safe Space at School: Exploring Power Dynamics, Bullying Locations, and Maladaptive Behaviors Among US Adolescents

**DOI:** 10.1007/s10964-025-02190-z

**Published:** 2025-05-12

**Authors:** Jacky Cheuk Lap Siu

**Affiliations:** The Chinese University of Hong Kong, Hong Kong, China

**Keywords:** Bullying, Bullying locations, Power imbalance, Adolescents, Latent class analysis

## Abstract

School bullying is not merely a random event but rather involves intricate power dynamics in each bullying event. However, less is known about whether this power asymmetry could lead to different spatial patterns of bullying within the school environment, contributing to distinct perceptions of the usage of social spaces within the school context defined by bullies and victims. Further, it remains unclear how differentiated bullying victimization experiences and power imbalances could jointly result in victims’ heterogeneous maladaptive behavioral responses. In response, this study was the first to conduct latent class analysis to identify subgroups of victims of bullying and compare these subgroups based on power differences with the bullies, locations of bullying, and their associations with distinct behavioral responses. This study used the adolescent sample from the 2022 School Crime Supplement (SCS) of the National Crime Victimization Survey (*n* = 1249; ages 12–18 years [mean: 14.3; SD: 1.85]; 45.8% male; 79.3% White, 9.8% Black, 2.5% Asian, and 8.4% other racial groups). Five victim classes emerged: “Marginal victims” (29.3%), “Outpowered victims” (28.2%), “Classroom-specific victims” (16.7%), “Hallway-specific victims” (16.8%), and “Pervasive victims” (9%). Results of logistic regression revealed that “Outpowered victims” and “Pervasive victims” were significantly more likely to exhibit maladaptive behaviors such as avoidance and fear, but only the “Pervasive victims” group showed an increased likelihood of carrying weapons to school, compared to the “Marginal victims” group. The findings suggest that bullying is intricately tied to power dynamics, with dominant-subordinate relationships reproduced in school spaces. Structured areas, such as classrooms and hallways, emerged as key bullying sites, challenging assumptions that bullying primarily occurs in unstructured spaces. This study reveals how power imbalances between adolescents shape their perception and use of school spaces, demonstrating that youth develop different behavioral responses based on their position in social hierarchies.

## Introduction

School bullying has been a chronic and ubiquitous problem for schools in the United States, characterized by repeated harmful actions and a power imbalance between the perpetrator and the victim (Olweus, 1993). While previous studies have identified bullying “hotspots” in both unstructured, less supervised areas like bathrooms (Cozma et al., 2015; Migliaccio et al., 2017) and structured, more visible spaces like classrooms and hallways (Francis et al., 2022; Perkins et al., 2014), research has yet to examine the factors that co-occur with the spatial distributions of bullying within the school environment. Undoubtedly, school bullying is often not random and involves intricate power dynamics and social interactions between various stakeholders. Bullies and victims, who have fundamentally different power positions, may have different perceptions of the “perceived” and “lived” spaces that shape the spatial patterns of bullying incidents (Garandeau et al., 2014). Nonetheless, it remains unclear whether the asymmetrical power structures between bullies and victims could contribute to different spatial patterns of bullying in schools, let alone how the interplay between these patterns and power imbalances could contribute to heterogeneous maladaptive developmental outcomes exhibited by the victims. This study is the first to examine the patterning of bullying locations and power imbalances with the utilization of latent class analysis. It also seeks to explore how the interactions between differentiated bullying experiences and exercise of social power are likely to have unique associations with heterogeneous maladaptive behavioral responses such as avoidance, fear of victimization, and weapon-carrying. These problematic behaviors could reflect adverse developmental changes in social-emotional adjustment and aggressive conflict coping strategies derived from bullying experiences among US adolescents.

### Correlations Between Bullying Locations and Power Imbalance

Bullying is defined as aggression that occurs repeatedly and over time in the context of an asymmetric power relationship (Olweus, 1993). It means that bullies tend to have a more powerful position in the group and choose victims who have less physical or social strength to minimize the cost of their bullying behaviors. More specifically, bullies tend to exploit their advantages, such as physicality, social status, and popularity, wealth, and age, to exert control or dominance over victims directly and indirectly. For direct forms of bullying, the bullies’ physical advantages can make it easier to intimidate, threaten, or even assault the less powerful victim. On the other hand, bullies with stronger social capital may dominate shared spaces to rally their peers against the victim (Garandeau et al., 2014; Kaufman et al., 2020).

Prior research has identified common “hotspots” where bullying incidents tend to take place. These hotspots include classrooms, playgrounds, hallways, locker rooms, and bathrooms, which imply that bullying hotspots are nearly everywhere—almost nowhere is immune/off-limits (Francis et al., 2022; Perkins et al., 2014). Interestingly, these studies have revealed that bullying is not limited to less supervised areas, such as bathrooms, but also occurs in more visible locations like classrooms, hallways, and lunchrooms, where there is relatively more adult supervision (Fram & Dickmann, 2012). Additionally, some studies have found that victims may experience higher levels of victimization in classrooms and hallways compared to concealed areas like bathrooms (Francis et al., 2022). Given the nearly ubiquitous spatial patterns of bullying locations, these findings may further suggest a potential correlation between bullying locations and the power imbalance between bullies and victims. On the one hand, victims may recognize the bullies’ physical advantage and take self-protective measures by avoiding concealed areas like bathrooms and locker rooms to minimize their proximity to potential victimization (Perkins et al., 2014).

On the other hand, it may also imply that bullies strategically perpetrate bullying against victims by engaging in covert behaviors when they realize they are being watched (Blosnich & Bossarte, 2011). Moreover, the relatively higher prevalence in overt areas may indicate the presence of other crucial roles in bullying events, such as bystanders and reinforcers. To minimize the risk of bullying events being witnessed by teachers and other adults in schools, bullies may assign other bullies, reinforcers, or even bystanders to act as lookouts, ensuring that their bullying remains concealed from adult supervision (Blosnich & Bossarte, 2011; Francis et al., 2022). However, bystanders who empathize with the victims may hesitate to intervene or report the bullying due to the power differential with the bullies (Saarento & Salmivalli, 2015; Thornberg et al., 2018). Consequently, the significant power held by bullies may embolden them to bully victims without restraint, even in more visible areas within schools. Therefore, the locations of bullying may be influenced by the power dynamics and social interactions among the different individuals involved in the bullying events (Migliaccio et al., 2017). Nevertheless, research investigating the correlation between the locations of bullying and the power imbalance between bullies and victims remains scarce to date.

### Theories Explaining the Correlation Between Bullying Locations and Power Imbalance

The current study introduces two theoretical frameworks that explain the correlation between bullying locations and the power imbalance between bullies and victims: Lefebvre’s (1991) theory of social production of space and Cohen & Felson’s (1979) routine activity theory. Initially developed in the context of capitalist production, previous studies have extended Lefebvre’s theory to education to analyze how space is created and defined within schools (Brett, 2024; Fataar & Rinquest, 2019). According to Lefebvre (1991), space is not merely an abstract and passive concept but can be actively constructed through human relations. At the core of Lefebvre’s theory of social production of space is the spatial triad, which consists of three interconnected perspectives on space: perceived, conceived, and lived. Perceived space refers to the physical aspects of space, while conceived space pertains to the intentional planning, design, and conceptualization of physical spaces by experts (Lefebvre, 1991). Lived space, also known as social space, refers to individual subjective experiences that may or may not align with the conceived and perceived spaces. Social space is constantly influenced and shaped by symbolic exchanges among individuals and other social actors within that space (Brett, 2024; Forsberg et al., 2024).

In the school context, perceived space refers to the physical buildings, grounds, and facilities of the school, while conceived space relates to the planning and design of physical spaces within the school (Forsberg et al., 2024). Lived space, on the other hand, encompasses the students’ subjective experiences that influence their perception of the conceived and physical space within the school (Brett, 2024). Regarding school bullying, different roles involved in bullying may have varying definitions and perceptions of space within school campuses. Specifically, bullies can actively shape the social and mental space of other bullying roles. For example, bullies may strategically engage in covert bullying behaviors or use their power to assign a reinforcer as a lookout to isolate their actions from being observed by authority figures (Fram & Dickmann, 2012). If intervention by school personnel is absent, successful bullying in more visible areas like classrooms and hallways can reinforce bullies’ belief that these overt areas are suitable for victimizing peers, perpetuating and intensifying bullying behavior within the school (Brett, 2024; Forsberg et al., 2024). From the perspective of victims, locations commonly referred to as “bullying hotspots,” such as restrooms, changing rooms, and hallways, can be experienced as negative social spaces that significantly compromise their sense of safety within the school environment (Forsberg et al., 2024). The prevalence of bullying in these areas can instill fear and anxiety in victims, leading to avoidance of specific places within the school or, in extreme cases, even school avoidance (Francis et al., 2022).

Another possible theoretical explanation can be attributed to Cohen & Felson’s (1979) routine activity theory. Originally developed to explain crime pattern changes in the late 1970s, routine activity theory offers an alternative perspective to traditional criminological theories by emphasizing how changes in individuals’ routine activities intersect with the presence or absence of criminal opportunities (Cho et al., 2017). It suggests that the occurrence of crime involves three main elements: motivated offenders, target suitability, and absence of capable guardianship (Cohen & Felson, 1979). In recent years, the routine activity perspective has been applied to explain school bullying in various studies (Cho et al., 2017; Cecen-Celik & Keith, 2019). These studies have generally supported this theory by showing that students’ exposure and proximity to motivated offenders, as well as the lack of protective relationships in schools (e.g., positive student-student and teacher-student relationships) and security measures (e.g., presence of CCTV), can significantly predict peer victimization (Nie et al., 2022). However, existing studies have primarily focused on examining how extracurricular routine activities relate to peer victimization at school, without considering the inherent power dynamics in bullying situations. As a result, the relationship between bullying locations and power imbalances between bullies and victims remains unexplored to date.

## Current Study

Despite the extensive research on examining bullying hotspots across school environments, a significant gap exists in understanding how the spatial patterns of bullying correlate with power imbalances between bullies and victims. Given the lack of empirical quantitative research using latent variable methods to illustrate the intersections between bullying locations and power imbalances among school-aged adolescents, the current latent class analysis is exploratory. This study improves upon and advances the current knowledge base on spatial patterns of bullying by exploring the correlations between power imbalances and bullying locations in schools and examining the relationship between subgroups of victims of bullying and different maladaptive behavioral responses (e.g., avoidance, fear of victimization, and weapon-carrying to school). Hence, the purpose of the current study is guided by two major research questions: What distinct patterns of bullying victimization exist among US adolescents based on the constructs of bullying locations and power imbalances between bullies and victims? How are these patterns of bullying victimization associated with behavioral responses such as avoidance behavior, fear of victimization, and weapon-carrying to school? It is hypothesized that rather than being a homogeneous group, victims of bullying would fall into relatively distinct clusters. Moreover, membership in these classes would be differentially associated with avoidance, fear of victimization, and weapon-carrying to school. As previous studies have shown that positive school characteristics could significantly mitigate peer victimization in school by reinforcing protective relationships and surveillance on school campuses and rectifying the power imbalances across different bullying roles, positive school climate and school security measures were controlled in the present study.

## Methods

### Data and Sample

This study utilized data from the 2022 School Crime Supplement (SCS) of the National Crime Victimization Survey (NCVS). The NCVS is an annual survey conducted by the U.S. Census Bureau on behalf of the Bureau of Justice Statistics. The NCVS employs a stratified cluster random sampling design, which initially involves randomly selecting geographic regions (Primary Sampling Units, PSUs). Subsequently, households within these selected PSUs are randomly chosen. SCS is a specific component focusing on gathering information about victimizations and student perspectives on crime and safety within educational settings. To gather the data, after completing the NCVS interview of the head of the household, youth aged 12–18 who were currently in primary or secondary education programs leading to a high school diploma (elementary through high school) were selected to participate in the SCS interview and were interviewed individually by a researcher. Youth who had dropped out, been expelled or suspended, or were temporarily absent from school were still eligible if they were enrolled and attending school at any time during the six months prior to the interview. This sampling methodology ensures that a representative sample of students provides insights into their experiences with crime and safety in schools.

Of the 12,335 respondents eligible for the 2022 SCS, 4837 (39.2%) completed the SCS interview. Among 4837 SCS respondents, 932 (19.3%) of them reported peer victimization experience and were subsequently asked questions related to the study variables, such as power imbalances and bullying locations. Among these 932 respondents, only individuals who provided valid responses, namely those who did not refuse to answer questions on all study variables, were included in the current study. The resulting sample included 880 individuals, of whom 45.8% (*N* = 403) were male. The sample was multiracial but predominantly White (79.3%; *N* = 698); 9.8% (*N* = 86) of them were Black, 2.5% (*N* = 22) of them were Asian, and 8.4% (*N* = 74) of them belonged to another race group. The average age was 14.3 (SD = 1.85) with a range of 12–18 and over half (*N* = 522, 59.3%) of the respondents were aged 14 or below, a critical developmental period marking the transition into early adolescence. Although the response rate for the 2022 SCS survey was relatively low, it is worthwhile to note that there is a lack of nationally representative data sources that comprehensively capture information on locations of bullying, power imbalances, and school security measures. Moreover, the SCS dataset has been extensively studied in previous empirical studies examining bullying behavior among US adolescents (Mindrila, 2019; Vidourek et al., 2016).

### Measurement

#### Locations of Bullying

The SCS asked victims of bullying, “Still thinking about all of the [time/times] that you were bullied during this academic year, where did the bullying occur? Did it occur… In: (1) classrooms, (2) hallways, (3) bathrooms or locker rooms, (4) gym, (5) lunchroom, (6) outside on school grounds, (7) on the way or from school such as on school bus, and (8) somewhere else?” The response options were dichotomized (1 = Yes, 0 = No). These eight items were included in the LCA models.

#### Power Imbalance

The respondents were asked the questions: “Was this person/ Were any of these people/ Was anyone in the group: 1) physically bigger or stronger than you, (2) more popular than you, (3) have more money than you, (4) have the ability to influence what other students think of you, (5) have more power than you in another way during this academic year?” A dichotomized (1 = Yes, 0 = No) option was given to the respondents. These five items were included in the LCA models.

#### School Security Measures

School security measures were assessed by 9 items including security guards and safety equipment (Jeong et al., 2013). “School security guards” measured whether there was a security guard or staff/adults supervising the hallways during this academic year. Sample questions of the variable “safety equipment” included “Does your school have 1) metal detectors, (2) locked entrances, visitor sign-in requirements, (3) student badges or picture identification, (4) locker checks, and (5) security cameras present at any time during this academic year?” In the survey, respondents were provided with options to indicate their response: (1 = yes, 2 = no, 3 = don’t know). These response options were recoded as follows: (1 = No, 2 = Yes), while treating “don’t know” options and any other residual responses as missing data.

#### Positive School Climate

Positive school climate was measured by an additive index of 11 items, consisting of two categories: interpersonal relationship and structure (Fisher et al., 2018). The assessment of interpersonal relationships was measured by 7 items. Sample questions asked during this academic year included: “There is a teacher or other adult at school who genuinely cares about you,” “Teachers treat students with respect,” “There is a fellow student at school who truly cares about you,” “listens to you when you have something to say,” and “believes in your potential for success?” The structure variable pertained to the school’s organizational framework and was assessed through four items. Participants were asked to indicate their level of agreement with statements such as “The school rules are fair,” “The punishment for breaking school rules is consistently applied regardless of one’s status,” “The school rules are strictly enforced,” and “Students are aware of the consequences for violating school rules?” All items were given a four-point Likert scale: (1) Strongly disagree, (2) Disagree, (3) Agree, and (4) Strongly agree. The total scores from the eleven items were used to represent the variable of positive school climate. The alpha of this scale is 0.663.

#### Behavioral Responses

##### Avoidance Behavior

School avoidance was assessed by whether the respondents avoided a particular place within the school campus or on school grounds in the past 6 months because they thought someone might threaten their personal safety. The places included in the SCS questionnaire were hallways, shortest routes to school, school entrance, restrooms, the cafeteria, the school parking lot, other places within the school, other places on school grounds, and the school bus stop. A binary response option (0 = No, 1 = Yes) was given to each of the questions, and ultimately, the avoidance measure was represented by a dichotomized variable indicating any positive response to these locations (1 = any positive response of avoiding responses, 0 = No) (Randa et al., 2019).

##### Fear of Victimization

Fear of victimization was gauged by three items assessing how frequently students feared that someone would attack or harm them on the school property, on a school bus or bus stop, and besides the times on school property or school grounds. A 4-point response option (1 = Never, 4 = Most of the time) was given to each of the questions. The fear of victimization variable was created by categorizing the responses into a dichotomous format, where any positive responses were assigned a value of 1, while no such responses were assigned a value of 0.

##### Weapon-carrying to School

To assess students’ weapon-carrying behavior, the SCS survey included three dichotomized items with response options of 0 (No) and 1 (Yes). These items aimed to determine whether students brought weapons, including guns, knives, or other weapons to school during the last school year. Positive responses to any of these items were coded as 1, indicating that the student reported carrying a weapon to school, while no responses were coded as 0, indicating the absence of weapon-carrying behavior. It is worth noting that in this study, approximately 5.5% (*N* = 48) of the sampled individuals reported carrying a weapon to school.

### Covariates

Sex was coded as Male (0), and Female (1).

Age was measured by the original unit in the respondents’ years.

Race was dummy coded (1 = Black only, 2 = Asian-only, and 3 = other racial group), treating the White-only group as the reference.

Household income was originally measured by a categorical variable with the response options including annual household income: (1) less than $5000, (2) $5000–$7499, (3) $7500–$9999, (4)$10,000–$12,499, (5) $12,500–$14,999, (6) $15,000–$17,499, (7) $17,500–$19,999, (8) $20,000–$24,999, (9) $25,000–$29,999, (10) $30,000–$34,999, (11) $35,000–$39,999, (12) $40,000–$49,999, (13) $50,000–$74,999, (15) $75,000–$99,999, (16) $100,000–$149,999, (17) $150,000–$199,999, and (18) $200,000 or more. It was later dummy coded (1 = $10,000–$24,999, 2 = $24,999–$99,999, and 3 = more than $100,000), with treating annual household income less than $10,000 as a reference. The descriptive statistics are presented in Table 1.

**Table 1:** Descriptive statistics. (*N* = 880)

Variables	Mean/%	SD
1. Difference in physicality	40.0%	0.49
2. Difference in popularity	50.8%	0.5
3. Difference in money	29.8%	0.48
4. Difference in ability to influence others	55.5%	0.5
5. Difference in more power in other ways	6.9%	0.26
6. Bullying victimization experience in classrooms	38.5%	0.49
7. Bullying victimization experience in hallways	39.4%	0.49
8. Bullying victimization experience in bathrooms or locker rooms	12.3%	0.33
9. Bullying victimization experience in gym	11.1%	0.32
10. Bullying victimization experience in lunchroom	23.9%	0.43
11. Bullying victimization experience in outside on school grounds	23.2%	0.43
12. Bullying victimization experience in on the way or from school such as on school bus	9.2%	0.29
13. Bullying victimization experience in somewhere else	3.1%	0.17
14. Male	45.8%	0.5
15. Age	14.3	1.85
16. White	79.30%	0.89
17. Household income	3.27	0.71
18. Security measure	3.8	1.78
19. Positive school climate	3.16	0.4
20. Avoidance behavior	13%	0.34
21. Fear of victimization	40%	0.49
22. Weapon-carrying to school	5.50%	0.23

### Analytic Strategy

Latent class analysis (LCA) was performed using Mplus 8.0. LCA is a person-centered approach that allows for the examination and modeling of heterogeneity within bullying locations and power imbalances between bullies and victims. Since Mplus 5.0, full information maximum likelihood (FIML) estimation was utilized as the default method to handle missing data in LCA models, allowing for unbiased parameter estimation by using all available information from observed data points rather than discarding incomplete cases. In this analysis, a categorical latent factor representing distinct patterns of responses derived from observed indicators was assumed. The determination of the best fitting model, including the number of classes, was based on several statistical criteria. These criteria included the Akaike information criterion (AIC), Bayesian information criterion (BIC), sample-size-adjusted Bayesian information criterion (ABIC), and the Lo-Mendell-Rubin adjusted likelihood ratio test (LMR LRT) (Lo, 2001). Generally, lower values of AIC, BIC, and ABIC indicate a better fitting model (Muthen & Muthen, 2010). A *p*-value smaller than 0.05 in the LMR LRT suggests that the model with the specific number of latent classes provides a significantly better fit to the data compared to the model with one fewer latent class (Lo, 2001).

Once the optimal number of latent classes was determined, individuals were assigned to classes based on their posterior probabilities, which represent the likelihood of belonging to each latent class. Subsequently, a set of binomial logistic regressions was conducted to explore the associations between different class memberships and behavioral responses, such as avoidance behavior, fear of victimization, and weapon-carrying to school. These analyses were performed using the poLCA R package in Jamovi 2.3.28. Demographic variables such as sex, age, race, and household income, and school-level variables such as positive school climate and security measures were controlled for in these analyses to account for their potential influence on the outcomes. Missing data were listwise deleted when classes were regressed on covariates, as the number of missing cases was small (*N* = 4).

## Results

### Latent Class Analysis

A series of LCAs with 1 through 7 classes were tested using eight indicators of locations of bullying and five indicators of power imbalances between bullies and victims. As shown in Table 2, as the number of classes increased from one to four, the AIC, BIC, and ABIC improved. The LMR LRT was significant across all models, with the exception of the comparison of model 7 to 6. These models suggest that the four-class solution was the best; nevertheless, this model resulted in an entropy value of 0.806, which was significantly lower than the models with three and five classes. Therefore, the models of three classes and five classes were further evaluated. Compared to the model of three classes, the five-class solution had a relatively lower BIC and a slightly lower entropy value. All five classes had an average posterior probability for membership above 0.90, except for Class 2, with 0.826. Thus, the model of five classes was chosen.

**Table 2:** Latent class analysis fit indices

Number of classes	AIC	BIC	ABIC	LMR LRT *p*-value	Entropy
1	13995.18	14119.45	14119.45	NA	NA
2	12932.34	13185.68	13017.36	< 0.001	1
3	12340.37	12722.76	12468.7	< 0.001	0.835
4	12100.11	12611.56	12271.75	< 0.001	0.806
5	12020.86	12661.37	12235.82	0.03	0.831
6	11981.27	12750.84	12239.54	0.001	0.818
7	12000.83	12899.45	12302.4	0.08	0.834

The bolded model indicates the model selected

*AIC* akaike information criterion, *BIC* bayesian information criterion, *LMR LRT* lo-mendell-rubin likelihood ratio test

**Fig. 1:**
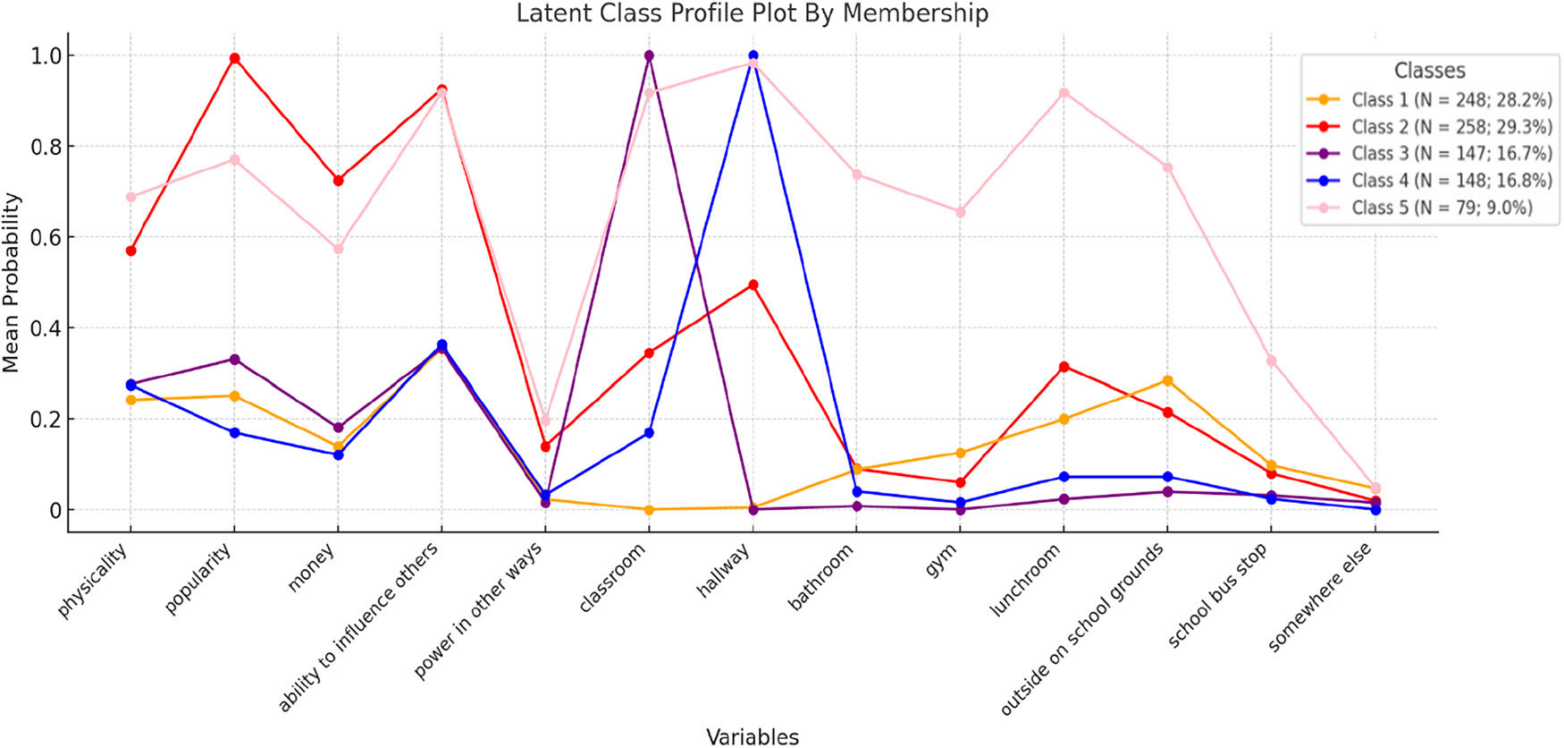
Item probability for each latent class. Class 1 (“Marginal victims”) comprises adolescents with a low probability of experiencing power imbalances with bullies and a low probability of being frequently victimized in school. Class 2 (“Outpowered victims”) consists of adolescents who have a high probability of experiencing power imbalances with bullies, but only a moderate probability of being bullied in the classroom and hallways. Class 3 (“Classroom-specific victims”) represents a group of adolescents with a low probability of experiencing power imbalances with bullies, but a high probability of being bullied specifically in the classroom. Class 4 (“Hallway-specific victims”) represents a group of adolescents who have a low probability of experiencing power imbalances with bullies, but a high probability of being bullied specifically in the hallways. Class 5 (“Pervasive victims”) consists of adolescents who exhibit a high probability of experiencing power imbalances with bullies and a high probability of being frequently victimized in any location within the school

Figure 1 displays the predicted probabilities for each class across the 13 indicators using the poLCA R package in Jamovi 2.3.28. The analysis resulted in five distinct classes with different patterns of responses. The largest class, Class 2 (*N* = 258; 29.3%), exhibited consistently high power imbalances with the perpetrators and only had a moderate probability of victimization in hallways (*p* = 0.47) and classrooms (*p* = 0.37). This class was labeled as “Outpowered victims.” The second largest class, Class 1 (*N* = 248; 28.2%), displayed consistently low probabilities of power imbalances with the perpetrators and only had a moderate probability (*p* = 0.28) of being victimized in other places on the school grounds. This class was labeled as “Marginal victims.” Class 3 (*N* = 147; 16.7%), labeled “Classroom-specific victims,” had a moderate probability (*p* = 0.37) of power imbalance in terms of the bullies’ ability to influence others and a high probability of victimization in classrooms. Class 4 (*N* = 148; 16.8%) was labeled as “Hallway-specific victims” because it had a moderate probability (*p* = 0.34) of power imbalance with the bullies in terms of their ability to influence others and had a high probability of being victimized in hallways. The fifth class, also the smallest class, Class 5 (*N* = 79; 9%), was labeled as “Pervasive victims” as it exhibited consistently moderate to high probabilities of power imbalances with the bullies and being victimized in almost all places within the school context.

### Behavioral Responses

Table 3 presents the results of a chi-square test of independence which revealed significant differences in avoidance behaviors, fear of victimization, and weapon-carrying across class memberships. Specifically, pervasive victims (Class 5) reported the highest levels of avoidance (38%), fear of victimization (66.6%), and weapon-carrying compared to all other groups, highlighting a distinct behavioral pattern among the most chronically victimized students. Furthermore, it is important to note that the study variables included in the regression analyses exhibited Variance Inflation Factors (VIFs) below 1.5. This suggests that multicollinearity was not a significant concern for these analyses. A set of binomial logistic regressions was performed to investigate the associations between different class memberships and behavioral responses, as presented in Table 4.

**Table 3:** Omnibus test of classes variables and behavioral responses

Variables	Classes					χ2	adj. *p*-value	Cramer’s V
	1	2	3	4	5			
Avoidance	8.9%_b_	17.4_b_	8.1%_a_	4.8%_b_	38%_b_	58.6	0.00	0.26
Fear of victimization	30.2%_a_	49.8%_b_	33.1%_a_	32.8%_a_	66.6%_b_	47.6	0.00	0.23
Weapon-carrying	4.40%_a_	3.9%_a_	6.7%_a_	2.7%_a_	21.2%_b_	22.6	0.00	0.16

Proportions with different alphabetic superscripts within a row significantly differ at adjusted *p* < 0.05. Class 1 refers to “Marginal victims”; Class 2 refers to “Outpowered victims”; Class 3 refers to “Classroom-specific victims”; Class 4 refers to “Hallway-specific victims”; Class 5 refers to “Pervasive victims”

**Table 4:** Multivariable logistic regression models

	Avoidance				Fear of victimization				Weapon-carrying			
	Estimate	SE	OR	OR95%CI	Estimate	SE	OR	OR95%CI	Estimate	SE	OR	OR95%CI
Latent class (Ref: Class 1)												
2	0.62**	0.28	1.86	0.06–1.18	0.81**	0.19	2.22	0.42–1.18	−0.23	0.46	0.79	−1.13–0.68
3	−0.67	0.45	0.51	−0.86–0.62	0.12	0.23	1.1	−0.33–0.56	0.49	0.46	1.62	−0.41–1.39
4	−0.12	0.38	0.89	−1.57–0.20	0.12	0.23	1.12	−0.32–0.57	−0.51	0.61	0.6	−1.70–0.67
5	1.69**	0.33	5.42	1.03–2.34	1.41**	0.28	4.1	0.86–1.96	1.33**	0.46	3.78	0.43–2.22
Race (Ref: White)												
Black	0.33	0.33	1.39	−0.31–0.99	0.62*	0.25	1.85	0.13–1.10	0.53	0.44	1.7	−0.33–1.40
Asian	−0.9	2.95	0.403	−2.94–1.13	−0.001	0.46	0.99	−0.90–0.90	−14.65	816.08	0	−1614–1584
Other race	0.28	0.33	1.33	−0.18–1.16	−0.16	0.24	0.86	−0.72–0.32	−1.77	1.03	0.17	−3.57–0.52
Household income (Ref: < $ 10,000)												
$10,000–$24,999	−0.61	0.6	0.54	−1.80–0.55	0.41	0.52	1.51	−0.60–1.43	0.08	0.97	1.08	−1.8–1.98
$24,999–$99,999	−1.01	0.5	0.36	−1.98–0.02	0.47	0.44	1.6	0.40–1.33	0.1	0.84	1.1	−1.54–1.75
> $100,000	−1.01	0.51	0.36	−1.99–0.01	0.26	0.45	1.3	−0.63–1.14	−0.19	0.86	0.83	−1.88–1.50
Age	−0.04**	0.06	0.96	−0.15–0.08	−0.12**	0.04	0.88	−0.20–0.04	0.28**	0.08	1.32	0.12–0.44
Female	0.23	0.22	1.26	−0.19–0.67	−0.06	0.15	0.94	−0.03–0.22	−0.1	0.32	0.9	−0.72–0.52
Positive school climate	−0.80**	0.26	0.45	−1.30–0.27	−0.67**	0.19	0.51	−1.04–0.30	−1.01**	0.36	0.36	−1.72–0.20
School security measure	−0.02	0.05	0.98	−0.13–0.09	−0.01	0.04	1	−0.08–0.06	0.04	0.08	1.04	−0.11–0.19

Class 1 refers to the “Marginal victims”; Class 2 refers to “Outpowered victims”; Class 3 refers to “Classroom-specific victims”; Class 4 refers to “Hallway-specific victims”; Class 5 refers to “Pervasive victims”

*p < 0.05, **p < 0.001

#### Avoidance Behavior

In comparison to the “Marginal victims” class, members of the “Outpowered victims” class showed significantly higher odds of avoiding places in the school context (b = 0.62, *p* < 0.05). Similarly, the “Pervasive victims” class demonstrated significantly higher odds of engaging in avoidance behavior (b = 1.69, *p* < 0.01) compared to the “Marginal victims” class. On the other hand, the memberships of the “Hallway-specific victims” class (b = −0.12, *p* > 0.05) and the “Classroom-specific victims” class (b = −0.67, *p* > 0.05) did not show a significant association with an increased risk for avoidance behavior when compared to the “Marginal victims” class.

#### Fear of Victimization

Compared to the “Marginal victims” class, both the “Outpowered” class (b = 0.81, *p* < 0.01) and the “Pervasive victims” class (b = 1.41, *p* < 0.01) exhibited significantly higher odds of experiencing fear of victimization. However, there was no significant difference in the risk of fear of victimization between members of the “Hallway-specific victims” class (b = 0.12, *p* > 0.05) or the “Classroom-specific victims” class (b = 0.12, *p* > 0.05) when compared to the “Marginal victims” class.

#### Weapon-carrying to School

In reference to the “Marginal victims” class, members of the “Pervasive victims” class displayed significantly higher odds of carrying a weapon to school (b = 1.33, *p* < 0.01). This suggests that individuals belonging to the “Pervasive victims” class are more likely to carry a weapon to school than those in the “Marginal victims” class. However, membership in the “Outpowered” class (b = −0.23, *p* > 0.05), “Hallway-specific victims” class (b = 0.49, *p* > 0.05), and “Classroom-specific victims” class (b = −0.61, *p* > 0.05) did not significantly differ from the “Marginal victims” class in terms of the likelihood of carrying a weapon to school.

### Sensitivity Analysis

Sensitivity analyses were conducted to evaluate the robustness of the findings and assess the influence of alternative modeling strategies. First, a comparison between the analytic sample (*N* = 880) and the excluded participants revealed significant demographic differences. Of the 12,335 initial SES eligible respondents, only 4837 answered the peer victimization items. Among these, 932 had responses to key LCA indicators, and the final analytic sample included 880 after accounting for residuals and refusals. The excluded participants were more likely to be male, older, Hispanic, and Asian. Second, alternative latent class models incorporating school security and positive school climate variables were considered. The model with school security variables resulted in a three-class solution, while the model including positive school climate variables produced a five-class solution. However, both models demonstrated poorer fit compared to the original model. Specifically, the entropy values (0.711 and 0.748, respectively) were lower than that of the original five-class model (0.831), and both AIC and BIC values were notably higher, indicating weaker model fit and class separation. These results suggest that the addition of school-level variables did not enhance the model’s explanatory power or interpretability.

Third, a latent class analysis was conducted on a subsample of adolescents aged 12–14. The resulting class structure was largely consistent with the full sample, with only one class shifting in contextual emphasis that changed from hallway-specific to lunchroom-specific victims. Class distributions and associated behavioral patterns remained stable, reinforcing the generalizability of the original model across early and full adolescence. Finally, alternative codings of the outcome variables were tested. The behavioral responses variables, including avoidance, fear of victimization, and weapon-carrying were recoded as continuous variables, and the linear regression results remained consistent with the original analyses and yet the effect size was much smaller than the original logistic regression results. For instance, the dominant power imbalance and pervasive victim groups continued to exhibit significantly higher levels of avoidance and fear, and only the pervasive victim group showed significantly elevated levels of weapon-carrying behavior. Given these considerations, binary coding and binominal logistic regression seem superior to enhance the interpretability and better aligns with this study’s focus on exploring the maladaptive behavioral adoption among bullying victims.

## Discussion

School bullying is a social event that involves inherent and yet often irreversible power imbalances between various bullying roles. Participants in bullying-related events may develop distinct interpretations and expectations of social spaces in schools. For instance, bullies may perceive certain areas as territories for asserting control, while victims might perceive these locations as threatening or unsafe. Despite extensive research identifying common school bullying “hotspots,” the influence of asymmetrical power structures on the spatial patterns of bullying incidents remains underexplored. The results of latent class analysis and binominal logistic regression highlight how adolescents navigate power imbalances within school environments, revealing that bullying is not just random aggression but reflects complex social hierarchies. The findings also indicate adolescents with different power positions and bullying experiences are associated with heterogeneous developmental outcomes such as psychosocial functioning and conflict coping responses by exhibiting different levels of maladaptive behavioral responses, including avoidance, fear of victimization, and weapon-carrying to school.

Prior studies have reported mixed findings regarding the locations of bullying incidents. For example, some studies (e.g., Cozma et al., 2015; Li et al., 2024) have emphasized that bullying is more prevalent in unstructured and unsupervised settings, such as restrooms or school corners, where adult presence is minimal. In contrast, other research (e.g., Francis et al., 2022; Perkins et al., 2014) has identified structured and supervised areas, such as classrooms and hallways, as hotspots for bullying. The present study aligns with the latter, identifying three distinct classes of location-specific bullying: the “Outpowered victims” (*N* = 258; 29.3%), “Hallway-specific victims” (*N* = 148; 16.8%), and “Classroom-specific victims” (*N* = 147; 16.7%) groups. These findings suggest that bullying incidents tend to occur more frequently in structured areas rather than in covert, unstructured locations. This finding may echo prior research which noted that teachers struggle to maintain constant supervision even in structured areas such as classrooms and hallways (Horton et al., 2023). This challenge is further compounded by scheduling constraints, as teachers often have additional responsibilities or need time for their own breaks, making it difficult to ensure consistent monitoring of these spaces. Hence, from the perspective of the routine activity framework, these structured spaces may lack “capable guardianship,” enabling bullying behaviors to persist even in seemingly monitored environments (Brooks & Cohen, 2020).

On the other hand, Lefebvre’s (1991) theory of the production of social space posits that the physical environment is not merely a neutral backdrop, but rather a dynamic social product shaped by the interplay of spatial practices, representations of space, and spaces of representation. According to this view, the process of spatialization involves the reproduction of dominant-subordinate social relations within the material environment. In other words, the physical development of spaces is inextricably linked to the social processes and power dynamics that unfold within and around them, imbuing these spaces with particular meanings and functions (Fram & Dickmann, 2012). This theoretical lens is particularly relevant to the current findings, which reveal a close relationship between the power imbalances experienced by students and the specific locations where bullying incidents take place (Horton et al., 2023). For instance, the “Hallway-specific victims” and “Classroom-specific victims” groups exhibited a high probability of victimization in these respective areas, which was correlated with a similar degree of power imbalance in terms of the ability to influence others. This suggests that the spatial configuration of the hallways and classrooms may serve as a mechanism for the manifestation of these power dynamics, requiring the less powerful students to adapt to the dominant-subordinate relationships within these spaces (Fram & Dickmann, 2012).

Similarly, the “Outpowered victims” group, characterized by significant power differentials across multiple dimensions (physicality, popularity, wealth, and social influence), was found to experience relatively higher levels of victimization in classrooms and hallways. These structured, more visible areas may represent spaces where bullies strategically leverage their advantages, potentially with the assistance of reinforcers who help conceal the bullying events from the observation of school personnel (Horton et al., 2023; Pouwels et al., 2016). In this way, the production of these specific school spaces can be seen as intimately connected to the reproduction of the power imbalances between the bullies and their victims.

Furthermore, while bystanders are often present in supervised areas such as classrooms and hallways, bullies may still engage in bullying by exploiting gaps in effective oversight or leveraging their dominant social status to discourage intervention. The superior power status of the bullies can deter bystanders from stepping in, as they may perceive the power dynamics as favoring the perpetrators (Thornberg et al., 2018). In doing so, bystanders may inadvertently reinforce the bullies’ sense of entitlement and dominance, shaping their positive lived experience of these spaces (bullying acts) and their mental understanding (mental space) of the acceptability of bullying in structured areas (Lefebvre, 1991). Consequently, such dynamics enable the perpetuation of bullying in more overt and visible areas. However, in contrast to prior research identifying restrooms as “hotspots” for bullying (Migliaccio et al., 2017), the present study found limited instances of victimization in these unstructured areas. This discrepancy could be attributed to victims actively avoiding these places due to a perceived lack of effective guardianship and a heightened sense of insecurity (conceived space) in these locations (Francis et al., 2022). Indeed, future qualitative studies are warranted to further explore these explanations in greater depth and to provide further insights into the interplay between spatial and power dynamics of school bullying.

A series of binomial logistic regressions were conducted to address the second research question regarding the association between patterns of bullying locations, power imbalances, and behavioral responses. The findings indicate that the “Pervasive victims” group exhibited the highest likelihood of engaging in avoidance, fear of victimization, and carrying a weapon to school, compared to the “Marginal victims” group. In contrast, while the “Outpowered victims” group also demonstrated a significantly elevated risk of avoidance and fear of victimization, they did not show an increased propensity for weapon-carrying. These results are consistent with Agnew’s (1992) General Strain Theory.

General Strain Theory posits that individuals who experience persistent and chronic strain are more likely to adopt maladaptive coping mechanisms, particularly when they lack access to constructive coping resources (Agnew, 1992, 2015). Within the context of bullying, the power differentials with the bullies create a persistent strain that is challenging to escape or rectify. This ongoing power imbalance can perpetuate feelings of helplessness, fear, and a sense of being trapped within a hostile environment (Bauman et al., 2023; Choi & Park, 2021). In the current study, the “Pervasive victims” group emerges as the most strained victim group, characterized by significant power differentials with bullies and repeated victimization across multiple school locations. Consequently, this heightened strain may induce the adoption of more aggressive behavioral responses such as weapon-carrying. Such actions may serve as self-protective measures to address perceived vulnerabilities or as retaliatory acts against their bullies (Pontes & Pontes, 2021). In contrast, members of the “Outpowered victims” group experience considerable strain related to power imbalances, but their behavioral responses are predominantly internalized, manifesting as avoidance or fear of victimization rather than outward aggression.

However, the magnitude of the strain experienced by this group may be insufficient to induce more aggressive behavioral responses. Unlike the “Pervasive victims” group, who endure chronic bullying across diverse school spaces, the “Outpowered victims” group lacks the compounded, persistent experienced strain necessary to escalate their coping strategies to more confrontational behaviors, such as weapon-carrying (Agnew, 2015). These findings highlight the importance of distinguishing between the types and intensity of strain in understanding how bullying victimization shapes behavioral adaptations. They also underscore the significance of applying a strain-based framework to contextualize the interaction between power imbalances, experienced strain, anticipated strain, and the corresponding behavioral responses among victimized students.

This study has several limitations that should be acknowledged. First, the cross-sectional nature of the SCS survey prevents it from making causal inferences on the associations between the classification of the five class groups and the observed behavioral responses. Longitudinal studies would be valuable in examining the stability of latent classes over time and determining the temporal sequence of the classification and its correlates. Further, given the low response rate and high exclusion criteria employed in this study, caution should be exercised when generalizing these findings to the broader population of U.S. adolescents. Second, all study variables were assessed using self-report measures. Since sexual-related harassment and bullying often occur in covert areas such as restrooms and locker rooms, victims of bullying may underreport their victimization due to social desirability bias (Espelage et al., 2016). In addition, nonresponse bias may be a potential concern, as students who experienced more severe forms of bullying or felt unsafe at school might have been less likely to respond. This could result in an underestimation of the prevalence and severity of bullying reported in this study. Third, the study focused on a sample of victims, encompassing all types of victimization. Future research should explore whether different subtypes of bullying victimization (e.g., racist bullying and sexist bullying) and various demographic samples, including gender and race, are associated with distinct patterns of bullying locations and power imbalances between victims and bullies. Last but not least, it is important to acknowledge that this study solely focuses on the types and prevalence of victimization that take place within different physical locations in schools. It does not encompass the victimization experience online, known as cyberbullying, during school hours or other times, and in different settings (Perkins et al., 2014).

Despite the limitations mentioned, the present study provides significant insights into anti-bullying programs and policies for school authorities. First, rather than relying solely on increased teachers’ supervision, which the findings suggest bullies may find ways to circumvent, a more holistic approach is warranted. One promising strategy is to broaden awareness to differentiated experiences among victims of bullying and mark the at-risk pupils who warrant particular help from practitioners and staff. School personnel should consider implementing a “buddy” system that pairs victims with peer mentors from higher grade levels, particularly targeting the “pervasive victims” group which exhibits a relatively high level of power imbalance with the bullies in terms of popularity and ability to influence others. This approach can help to directly address the power imbalances that enable bullying by providing victims with a source of social support and protection and help to reduce the sense of marginalization in schools (Barboza et al., 2009; Tzani-Pepelasi et al., 2019). Empowering these at-risk victims through these supportive peer relationships may bolster their sense of security and ability to resist or report bullying incidents, even in structured areas where bullies have been shown to persist in their harmful behaviors. Moreover, mental health professionals and social work practitioners should provide regular access to mental health counseling or therapeutic support to at-risk students and their families with tailored workshops addressing specific behavioral problems such as avoidance and fear of victimization (Gaffney et al., 2021). Additionally, programs that actively engage bystanders to intervene when witnessing bullying should also be developed (Bezerra et al., 2023; Burger et al., 2022). Anonymous reporting mechanisms and rewarding bystander action can harness the broader peer network to disrupt the social dynamics that allow bullies to dominate certain spaces (Novick & Isaacs, 2010). Last but not least, the findings suggest that bullies may exploit power imbalances to target victims in both visible and unsupervised school areas, highlighting a gap in traditional monitoring methods, such as the absence of consistent adult supervision in structured areas. Surveillance systems (e.g., CCTV) and patrol robot equipped with AI technology, using natural language processing and behavioral analysis, may help to monitor high-traffic areas like hallways and cafeterias, detecting subtle bullying behaviors, such as verbal aggression or social exclusion with high accuracy (Sidhu & Sidhu, 2025). Implementing these surveillance systems can enable real-time monitoring, map the bullying hotspots, and maintain a consistent guardianship to deter the bullies from victimizing their peers.

## Conclusion

Despite extensive research having been conducted to identify common bullying “hotspots”, little attention has been placed on examining how power dynamics influence the spatial patterns of bullying in schools. Additionally, limited research has explored whether distinct bullying experiences combined with significant power imbalances jointly contribute to unique associations with maladaptive behavioral responses. The current study is the first to explicitly examine the intersection between power imbalances and bullying locations, and to explore how these combined patterns shape maladaptive behavioral responses such as avoidance, fear of victimization, and weapon-carrying. The five-class model from the LCA analysis identified distinct patterns of bullying locations and power imbalances. The findings highlight that bullying incidents can happen in various contexts, including classrooms and hallways, which are typically assumed to be closely supervised by adults. These findings dissect how dominant-subordinate relationships are reproduced within school spaces, providing insights for creating safer environments by addressing both locations and power dynamics that enable bullying, ultimately supporting more equitable educational settings. More importantly, the findings in this study add to the field of school bullying by describing two “at-risk” victim groups, namely Outpowered victims and Pervasive victims, with different levels of overlap between power imbalance and bullying victimization experiences within the school context. It helps to expand the existing literature on school bullying by underscoring that victims with intersectional vulnerability could experience critical adverse developmental outcomes such as aggravated psychosocial functioning and aggressive conflict coping adoption, as embodied by the three maladaptive behavioral responses in the present study.
